# A novel highly specific qPCR assay for distinguishing HBV cccDNA from rcDNA following bisulfite pretreatment

**DOI:** 10.1128/jvi.00961-26

**Published:** 2026-08-14

**Authors:** Ning Sun, Zhili Wang, Zach Heimer, Chris Wenner, Selena Y. Lin, Heidi Y. Ding, Roman Kubas, You-Yu Liu, Eliza Qiu, Hsin-Ni Liu, Hsu-Chin Hung, Wenya Huang, Tianlun Zhou, Daryl T.-Y. Lau, Ying-Hsiu Su, Haitao Guo

**Affiliations:** 1 Department of Microbiology and Molecular Genetics, and Cancer Virology Program, UPMC Hillman Cancer Center, University of Pittsburgh School of Medicine 12317, Pittsburgh, Pennsylvania, USA; 2 JBS Science Inc, Doylestown, Pennsylvania, USA; 3 The Baruch S. Blumberg Institute, Translational Medical Science 480639, Doylestown, Pennsylvania, USA; 4 Department of Medical Laboratory Science and Biotechnology, National Cheng Kung University 541413, Tainan, Taiwan; 5 Liver Center, Beth Israel Deaconess Medical Center, Harvard Medical School 1811 , Boston, Massachusetts, USA; Dartmouth College Geisel School of Medicine, Hanover, New Hampshire, USA

**Keywords:** HBV, cccDNA, bisulfite conversion, qPCR

## Abstract

The persistence of covalently closed circular DNA (cccDNA) underlies chronic hepatitis B virus (HBV) infection and remains as the major barrier to achieving a functional cure. An accurate and sensitive quantification of cccDNA is critical for evaluating therapeutic strategies. The conventional quantitative PCR (qPCR) lacks specificity to distinguish cccDNA from viral relaxed circular DNA (rcDNA) and other replicative intermediates, while nuclease pretreatment (e.g., plasmid-safe ATP-dependent nuclease, exonucleases I/III, or T5 exonuclease) may either incompletely digest rcDNA and/or risk over-digesting cccDNA. Here, we evaluated a novel patented bisulfite conversion-based cccDNA (BSC-cccDNA) qPCR platform that employs bisulfite treatment to disrupt rcDNA complementarity, followed by qPCR with primer sets targeting the rcDNA gap region, to enable selective amplification of cccDNA. Using synthetic HBV DNA controls and DNA samples extracted from HBV stable cell line and HBV-infected cells, we demonstrate that the BSC-cccDNA qPCR assay achieves high specificity, with tolerance of up to 10^9^ rcDNA copies and sensitivity to 10 cccDNA copies per reaction. Comparative analyses with sensitive digital PCR (dPCR), cross-gap qPCR, and Southern blot further validated the sensitivity and accuracy of the BSC-cccDNA assay. These findings establish the BSC-cccDNA qPCR assay as a reliable tool for standardized cccDNA measurement and highlight its potential for both basic research and clinical applications.

**IMPORTANCE:**

Accurate quantification of hepatitis B virus (HBV) covalently closed circular DNA (cccDNA) is critical for understanding viral persistence and evaluating curative therapeutic strategies. However, current PCR-based assays lack sufficient specificity to distinguish cccDNA from relaxed circular DNA (rcDNA). Enzymatic pretreatment approaches to remove rcDNA introduce variability, risk incomplete digestion, and potential cccDNA loss, thereby compromising quantitative accuracy. We evaluated a novel bisulfite conversion-based cccDNA (BSC-cccDNA) qPCR assay that chemically disrupts rcDNA complementarity, enabling highly specific cross-gap amplification without reliance on nuclease digestion. Using synthetic substrates, cell-derived HBV DNA, and infected hepatocyte models, we demonstrate that this assay tolerates up to 10⁹ rcDNA copies while detecting as few as 10 cccDNA copies, with performance validated against Southern blot and dPCR. By improving both specificity and quantitative reliability, the BSC-cccDNA platform provides a potential standardized tool for accurate cccDNA measurement in basic research and therapeutic development.

## INTRODUCTION

Chronic hepatitis B virus (HBV) infection remains a significant global health problem affecting an estimated 296 million people worldwide and leading to nearly a million deaths annually from associated liver diseases, including cirrhosis and hepatocellular carcinoma (1). Current antiviral therapies, primarily nucleos(t)ide analogs (NUCs), are highly effective at suppressing viral replication but rarely lead to a complete virological cure due to their inability to eliminate the persistent, episomal form of the viral genome: the covalently closed circular DNA (cccDNA) (2, 3). The cccDNA resides in the nucleus of infected hepatocytes and serves as the stable transcriptional template for all viral RNAs, thereby acting as the molecular reservoir responsible for viral persistence and potential reactivation upon cessation of treatment (4–6).

An accurate and sensitive quantification of intrahepatic cccDNA is essential for both understanding the mechanisms of HBV persistence and for the preclinical and clinical evaluation of novel antiviral drugs targeting cccDNA. As reported previously, current methods, such as Southern blot and various quantitative PCR (qPCR)-based assays, including the conventional qPCR and digital PCR (dPCR), are hampered by inherent technical challenges and limitations to quantify cccDNA. Southern blotting directly visualizes cccDNA but is labor-intensive and insensitive for quantification (7). qPCR is more sensitive and quantitative but cannot reliably distinguish cccDNA from rcDNA due to generation of a false PCR product after two amplification cycles from rcDNA, even when a cross-gap primer set is used (8). This false PCR product derived from rcDNA compromises the specificity of cross-gap qPCR assay to 25% in theory (8). To increase the specificity of cross-gap cccDNA PCR assay, various nuclease pretreatments, such as plasmid-safe ATP-dependent nuclease (PSAD), DNA heat-denaturation plus PSAD, exonucleases I and III (Exo I/III), and T5 exonucleases (T5 exo), are employed to degrade non-cccDNA templates (e.g., rcDNA, other viral DNA replicative intermediates, and integrated HBV DNA) (9–11). However, these pretreatment approaches exhibit variability and potential of incomplete rcDNA removal or cccDNA over digestion. Thus, attempts for standardization of cccDNA assay, including cross-validations of distinct cccDNA extraction and quantification protocols using infected tissues and cell cultures, could only recommend for optimizing, controlling, and validating cccDNA measurements according to the sample type if cross-gap qPCR assays are to be used (12).

**Fig 1:**
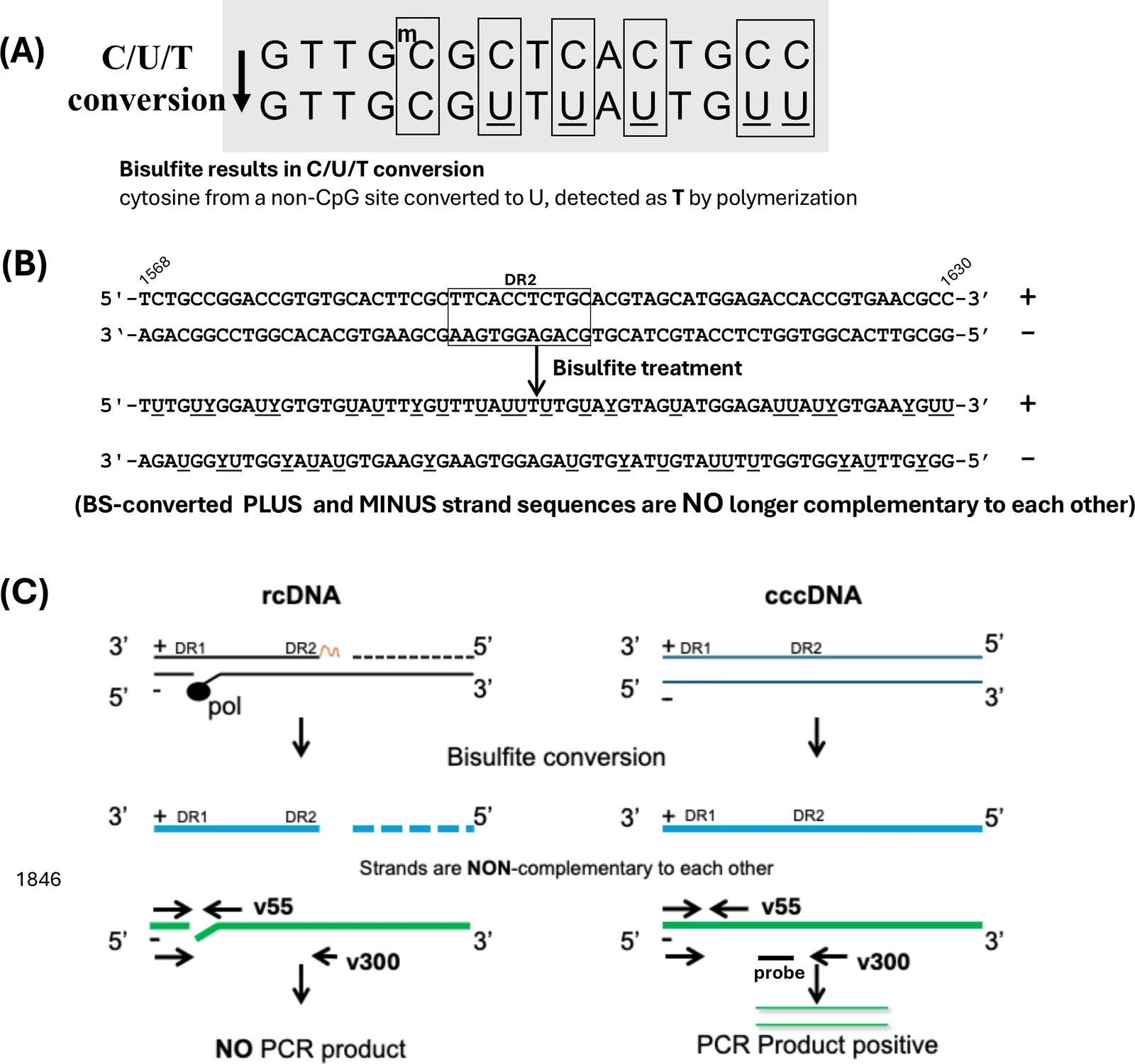
Elimination of PCR false-positive products derived from rcDNA by bisulfite conversion (BSC) and cross-gap primer design. (**A**) Bisulfite conversion (BSC) converts unmethylated cytosines to thymidines. (**B**) Example illustrating disruption of double-stranded DNA complementarity by BSC. The sequence of an HBV DNA fragment (nt 1568–1630, GenBank no. U95551.1) is shown as an example. The DR2 motif is indicated by a rectangle. Bisulfite treatment chemically converts unmethylated cytosines (C) to uracil (U) (underlined), thereby abolishing canonical base pairing with guanine. At CpG sites, cytosines may be either methylated or unmethylated; thus, “Y,” underlined, denotes C (methylated, retained as C) or U (unmethylated, converted from C) depending on methylation status. (**C**) Schematic illustrating specific detection of cccDNA following bisulfite conversion (BSC) in combination with cross-gap PCR primer design targeting the minus strand of the DR1 region. The BSC-cccDNA v55 assay (55 bp amplicon, nt 1792–1846, GenBank no. U95551.1) and a longer-amplicon (300 bp, nt 1547–1846, GenBank no. U95551.1) selectively amplify cccDNA after bisulfite-mediated disruption of rcDNA complementarity using minus-strand–specific cross-gap primer sets spanning the rcDNA gap region.

To address these challenges, we evaluated a novel, patented, commercially available cccDNA qPCR assay platform that employs bisulfite conversion (BSC) to chemically modify DNA to disrupt double-stranded DNA complementarity, thereby enabling strand-specific amplification (Fig. 1) (13). In combination with a minus-strand-specific cross-gap primer design, the BSC-based cccDNA assay is theoretically capable of eliminating rcDNA amplification, resulting in highly specific cccDNA quantification. We assessed the sensitivity and specificity of two BSC-based cccDNA assays: a short-amplicon (55 bp, nt 1792–1846, GenBank no. U95551.1) BSC-cccDNA v55 assay targeting the DR1 region and a longer-amplicon (300 bp, nt 1547-1846, GenBank no. U95551.1) BSC-cccDNA v300 assay targeting the DR2-DR1 region using synthetic rcDNA and cell-derived HBV core DNA. Assay performances were compared with the gold-standard Southern blot hybridization, conventional cross-gap qPCR, and dPCR assays with various exonuclease pretreatments. We further evaluated the assay performance using Hirt DNA and total DNA extracted from HBV-stable cell line, HBV-infected primary human hepatocytes (PHH), clinically relevant hepatocyte models, and liver biopsy specimens from chronic hepatitis B (CHB) patients.

## RESULTS

### Bisulfite conversion provides a high specificity of a cross-gap cccDNA PCR from rcDNA

To assess the specificity to detect cccDNA from rcDNA by the combination of bisulfite conversion (BSC) treatment and minus-strand-specific cross-gap primer design for DR1 region (nt. 1792–1846), we used a synthetic rcDNA (srcDNA) substrate reminiscent of the fragment (nt. 1733–1912) containing the nick/flap region on the minus strand, which was assembled by three single-stranded oligonucleotides (Fig. 5). To cover a wide dynamic range, 10-fold serial dilutions of srcDNA (10^7^–10^9^ copies) were mixed with 100 copies of H100 cccDNA plasmid control (H-srcDNA, detailed in Materials and Methods) or IDTE buffer control (srcDNA) subjected to BSC and analyzed using BSC-cccDNA qPCR v55 assay.

As shown in Table 1 (v55, left panel), the H100 cccDNA control was consistently detected at 150–180 copies in the presence of up to 10⁹ copies of srcDNA. No non-specific amplification was observed when rcDNA input reached 10^9^ copies. These results indicate that BSC coupled with DR1 cross-gap PCR primer design provides high specificity for cccDNA amplification, effectively excluding srcDNA at input levels up to 10⁹ copies.

**TABLE 1:** Specificity assessment of the BSC-cccDNA qPCR assay

BSC-cccDNA v55						BSC-cccDNA v300		
srcDNA and H100^*a*^			core DNA and H100^*a*^			core DNA and H100^*a*^		
srcDNA + H100	Input copies^*b*^^,*^ (mean ± SD)	cccDNA copies (mean ± SD)	Core + H100	Input copies^*b*^^,#^ (mean ± SD)	cccDNA, cp/μL (mean ± SD)	Core copies^*b*^^,#^	cccDNA (Core), cp/μL (mean ± SD)	cccDNA (Core + H100), cp/μL (mean ± SD)
**H-srcDNA-E9**	(6.48 ± 2.77) E8	(1.65 ± 0.43) E2	**H-Core-E8**	(1.54 ± 0.34) E8	(1.63 ± 0.07) E5	**1 × E8**	8,160 ± 380	12,200 ± 580
**H-srcDNA-E8**	(1.39 ± 0.19) E8	(1.57 ± 0.20) E2	**H-Core-E7**	(1.75 ± 0.01) E7	(1.07 ± 0.03) E4	**1 × E7**	731 ± 34	932 ± 81
**H-srcDNA-E7**	(3.91 ± 0.31) E6	(1.77 ± 0.58) E2	**H-Core-E6**	(8.58 ± 0.48) E5	(1.28 ± 0.28) E3	**1 × E6**	115 ± 17	310 ± 50
**H100 (H)**	(1.05 ± 0.16) E2	(1.59 ± 0.19) E2	**H-Core-E5**	(6.53 ± 0.02) E4	(2.08 ± 0.30) E2	**1 × E5**	21 ± NA	137 ± 41
**srcDNA-E9**	(6.31 ± 1.52) E8	BLOD	**H-Core-E4**	(6.33 ± 1.08) E3	(7.68 ± 2.78) E1	**1 × E4**	44 ± NA	140 ± 40
**srcDNA-E8**	(1.60 ± 0.12) E8	BLOD	**H-Core-E3**	(1.34 ± 0.06) E3	(6.35 ± 1.07) E1	**1 × E3**	BLOD	245 ± 40
**srcDNA-E7**	(7.88 ± 0.43) E6	BLOD	**H100 (H**)	(5.62 ± 1.79) E1	(6.62 ± 1.61) E1	**H100 (H**)	NA	353 ± 114
**IDTE**	BLOD	BLOD	**Core-E8**	(1.89 ± 0.01) E8	(8.34 ± 0.53) E4	**IDTE**	BLOD	BLOD
			**Core-E7**	(1.80 ± 0.06) E7	(8.36 ± 0.27) E3			
			**Core-E6**	(1.41 ± 0.17) E6	(1.03 ± 0.07) E3			
			**Core-E5**	(5.55 ± 0.30) E4	(1.34 ± 0.30) E2			
			**Core-E4**	(6.68 ± 0.43) E3	BLOD			
			**Core-E3**	(8.75 ± 1.50) E2	BLOD			
			**IDTE**	BLOD	BLOD			

^*a*^ H100 input: 100 copies; BLOD: below limit of detection; NA: not available.

^*b*^ HBV input quantities were determined by HBV 1778 (*) or HBV 1655 (^#^) qPCR assays.

We next evaluated the specificity using a biologically derived rcDNA preparation, HBV core DNA. Core DNA isolated from HepAD38 (tet-) cells was subjected to BSC and analyzed in the presence (H-Core) or absence (Core) of the H100 cccDNA control. As summarized in Table 1 (v55, right panel), approximately 64–77 copies of cccDNA signals were detected by BSC-cccDNA v55 qPCR assay from 100 copies of H100 alone, as well as from 100 copies of H100 reconstituted with core DNA at input levels up to 10⁴ copies (H-Core-E4) per reaction. Additional signals were observed when core DNA input reached ≥10⁵ copies, with signal intensity increasing proportionally across 10-fold dilutions starting from 10⁶ copies (H-Core-E6). Similar specificity was observed with core DNA alone. In the absence of H100 cccDNA control, the signal was detected when core DNA input reached 10^5^ copies, and the signal increased proportionally with higher core DNA inputs. Compared with the higher specificity observed with srcDNA, these results suggest that the complexity of core DNA qPCR may be due to the heterogeneous HBV capsid-associated DNA replicative intermediates in addition to rcDNA. This heterogeneity of HBV DNA species may influence the specificity of BSC-cccDNA v55 assay, and the detected false positive signals might originate from non-rcDNA species enclosed within core particles consist of approximately 0.1% of the total input HBV DNA.

To investigate whether incorporating an internal probe could further improve assay specificity, we tested a probe-based BSC-cccDNA v300 assay, which targets a ~300 nt region of the minus strand (nt 1547–1846) (Fig. 1). The BSC-cccDNA v300 assay was applied to aliquots of the bisulfite-converted HBV core DNA samples used in Table 1 (v300) in the presence (Core +H100) or absence (Core) of the cccDNA control H100. A detectable amplification signal, although below the assay’s limit of quantification (10 copies per reaction), was observed from core DNA alone at input levels of 10^4–5^ copies. In addition, approximately 100 copies of cccDNA were detected in 10^6^ copies of core DNA input. Notably, lower signal quantities were obtained with the v300 qPCR compared to the v55 qPCR from similar core DNA inputs. This difference is expected given DNA fragmentation induced by BSC, as shorter amplicons are more efficiently amplified from fragmented templates and, therefore, provide higher quantity output. Comparable cccDNA signals were observed with the H100 control alone and in the presence of 10⁵ copies of core DNA. Although the signal was detected when 10^6^ copies input core DNA, signal intensity did not increase proportionally with increasing input until 10^7^. Together, these results demonstrate that the BSC-cccDNA v300 assay exhibits slightly higher specificity for cccDNA in the presence of core DNA compared with the v55 assay.

### Sensitivity and linearity of BSC-cccDNA assays

**Fig 2:**
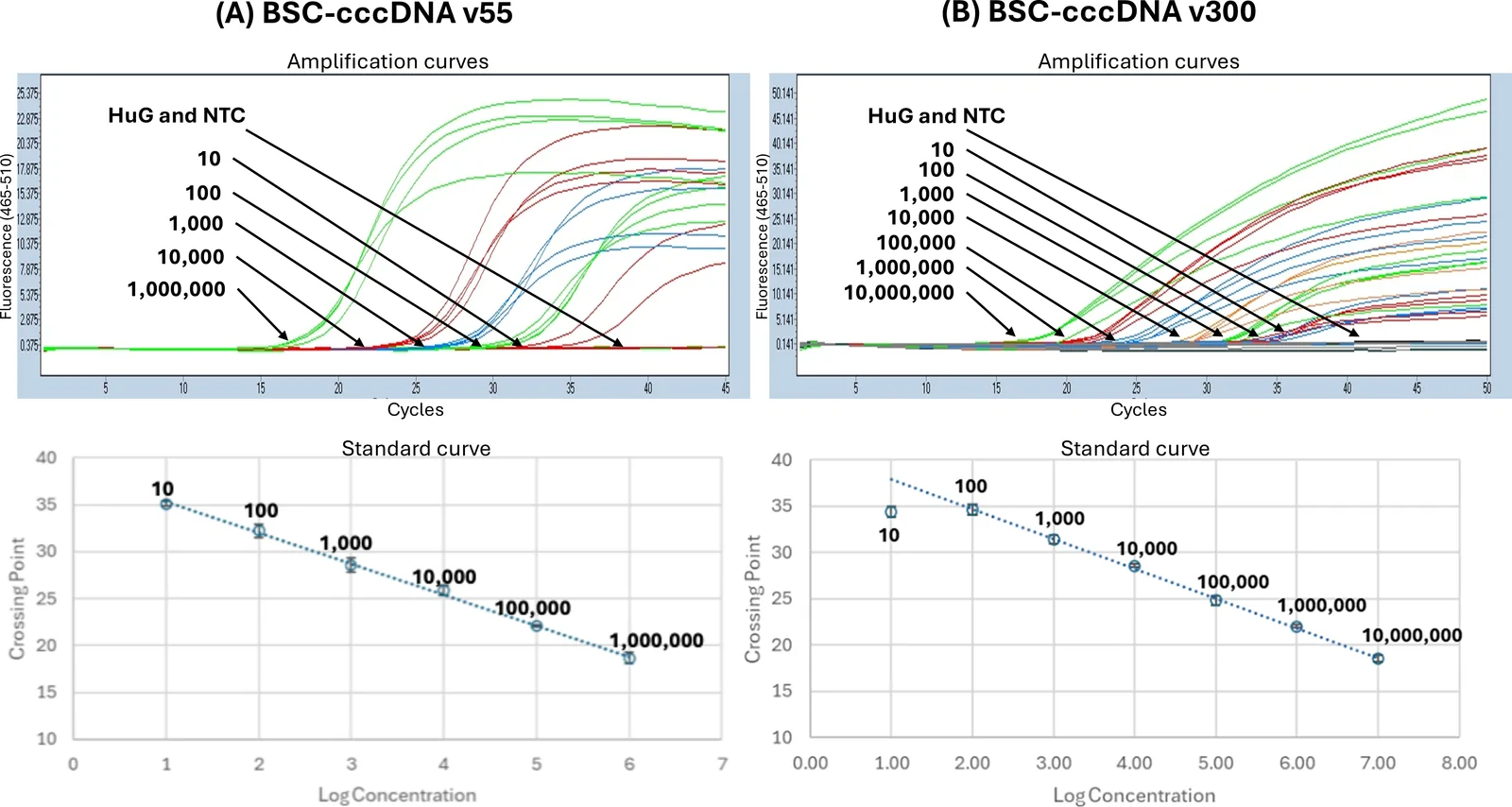
Sensitivity (limit of detection, LOD) and linearity (limit of quantification, LOQ) of BSC-cccDNA assays v55 (**A**) and v300 (**B**).

To determine the assay sensitivity, we used 10-fold serial dilutions of cccDNA control H100 spiked into 10 ng human genomic DNA (gDNA), followed by BSC and quantitated by BSC-cccDNA v55 (55 bp) and BSC-cccDNA v300 (300 bp) assays, as described in Materials and Methods. The amplification curves and standard curves with error bars are shown in Fig. 2. As indicated by arrows, the short-amplicon BSC-cccDNA v55 assay reliably detected as few as 10 input copies of H100, with linear quantification from 10 to 10^6^ copies. The BSC-cccDNA v300 assay remains sensitive to 10 input copies (limit of detection) but requires >100 input copies for reliable quantitation with a linearity from 10^2^ to at least 10^7^ copies.

### Bisulfite conversion does not compromise cccDNA quantification by short amplicon qPCR assay

Bisulfite conversion is known to be potentially detrimental to DNA integrity by (i) converting double-stranded DNA into labile single-stranded DNA and (ii) introducing nicks into the DNA backbone (14, 15). We, therefore, sought to determine whether the BSC process impacts the cccDNA templates used for PCR assay when a short amplicon PCR (BSC-cccDNA v55) was used as compared with a cross-gap dPCR assay.

Hirt DNA was extracted from five independent HBV-infected primary human hepatocyte cultures (PHHs) (IDs #1–5) and treated with T5/T7 exonuclease, as described in Materials and Methods. The treated HBV DNA samples were first quantified using the HBV1655 qPCR assay (Table S1), then analyzed in parallel either by dPCR or subjected to bisulfite conversion followed by BSC-cccDNA v55 qPCR for cccDNA quantification.

As summarized in Table 2, cccDNA quantities measured by dPCR and BSC-cccDNA v55 assays were generally comparable across samples. For example, sample 5 yielded nearly identical mean values by dPCR (279 copies) and BSC-cccDNA v55 (259 copies), while samples 1 and 3 showed less than twofold differences between the two assays, although larger discrepancies were observed in samples 2 and 4, particularly sample 2. Overall, these results indicate that bisulfite conversion did not appear to significantly reduce the availability of the templates for accurate cccDNA quantification when using BSC-cccDNA v55 short amplicon assay.

**TABLE 2:** Comparison of cccDNA quantitation by BSC-cccDNA v55 qPCR and dPCR assays

ID	Input HBV copies^*a*^ (mean)	cccDNA copies (mean ± SD)	
		dPCR	BSC-cccDNA
1	892	525 ± 11.7	281 ± 343
2	1,708	320 ± 6.4	1,050 ± 553
3	1,328	294 ± 22.5	404 ± 495
4	1,356	290 ± 10.6	608 ± 225
5	361	279 ± 8.5	259 ± 121

^*a*^ HBV input quantities were determined by HBV 1655 qPCR assays. Samples were analyzed in duplicate, and results were accepted when the two Cp values differed by less than 0.5 cycles. Samples with Cp values differing by more than 0.5 cycles were reanalyzed in triplicate, and the mean was calculated from the measurements differing by less than 0.5 cycles.

### Comparison of BSC-cccDNA qPCR and other cccDNA qPCR assays using Southern blot analysis as reference

BSC-cccDNA qPCR assays effectively distinguish bisulfite-converted srcDNA from plasmid cccDNA controls; hence, it is important to evaluate its applicability for quantifying biological samples in comparison with currently used cccDNA detection methods. To further assess the performance of the BSC-cccDNA qPCR assay, we employed the gold-standard Southern blot hybridization to visualize the various forms of HBV DNA.

**Fig 3:**
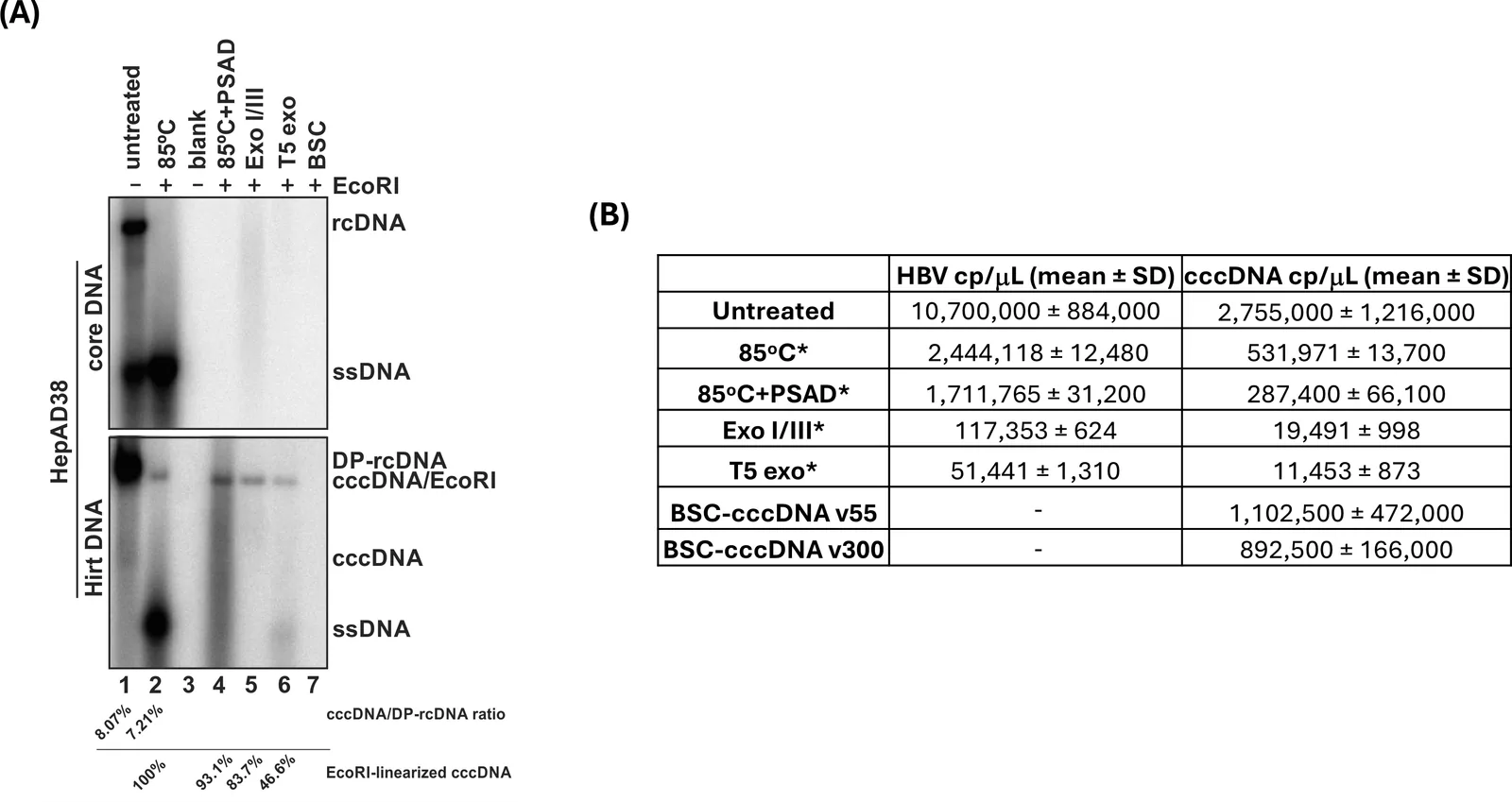
Comparison of HBV cccDNA quantification by Southern blot and qPCR-based methods. HBV cytoplasmic core DNA (**A**, top) and Hirt DNA (**A**, bottom) extracted from induced HepAD38 cells were either untreated or subjected to the indicated pretreatments, followed by analysis by Southern blot hybridization (**A**) and by cross-gap PCR or BSC-cccDNA qPCR assays (**B**). EcoRI digestion was applied only to pretreated Hirt DNA prior to gel loading for Southern blot analysis. The intensities of indicated HBV Hirt DNA bands on Southern blots were quantified densitometrically after subtracting the background signal from each lane, and the relative levels are presented. An aliquot of the same pretreated Hirt DNA samples without EcoRI digestion was further analyzed by cross-gap PCR or by BSC-cccDNA v55 and v300 qPCR assays. Total HBV DNA was quantified using the HBV1655 qPCR assay (B, middle column). cccDNA levels (B, right column) were measured by either the BSC-cccDNA qPCR assays or by conventional cross-gap PCR denoted by “*”.

First, we tested cytoplasmic HBV core DNA extracted from HepAD38 cells after 7 days of induction for the following treatments: (i) 85°C heat denaturation with or without PSAD; (ii) Exo I/III digestion; (iii) T5 exo digestion, or (iv) BSC, followed by Southern blot analysis. HepAD38 is a HepG2-derived HBV stable cell line that inducibly expresses viral genome upon withdrawal of tetracycline and is widely used for studies of HBV replication and cccDNA biology (16). Cytoplasmic HBV core DNA consists primarily of replication intermediates, such as single-stranded DNA (ssDNA) generated during reverse transcription, and rcDNA, which is further synthesized from ssDNA and serves as the major precursor of cccDNA formation through intracellular recycling in HepAD38 cells. As shown in the top panel of Fig. 3A, rcDNA was heat denatured into ssDNA, resulting in a proportional increase of ssDNA hybridization signal (lanes 1–2); ssDNA was further removed by PSAD digestion (lane 4). Furthermore, Exo I/III or T5 exo directly digested core DNA (lanes 5–6), and BSC treatment resulted in undetectable hybridization signal due to BSC-induced HBV DNA sequence alterations that eliminated complementarity to the probe (lane 7).

Next, Hirt DNA extracted from HepAD38 cells after 14 days of induction was either untreated or subjected to the above treatments. Hirt extraction is a specialized method used to isolate low-molecular weight extrachromosomal DNA species, including viral episomal DNA and mitochondrial DNA, while removing the majority of high-molecular weight host chromosomal DNA (17). HBV Hirt DNA extracts mainly contain a mixture of cccDNA and deproteinated rcDNA (DP-rcDNA), which is a protein-free rcDNA species devoid of the covalently attached viral polymerase and a putative intermediate during cccDNA formation (7, 18–20). The processed samples were first quantified using the HBV1655 qPCR assay, and then analyzed by Southern blot, conventional cross-gap qPCR, or BSC-cccDNA qPCR v55 and v300 quantitation, as summarized in Fig. 3B. Consistent with HBV DNA quantification by HBV1655 qPCR assay, untreated Hirt DNA samples exhibited the strongest hybridization signals, whereas no detectable signal was observed from DNA after BSC due to the chemical alteration of HBV sequences (Fig. 3A, lower panel, lanes 1 and 7). Notably, incubation at 85°C denatured DP-rcDNA into ssDNA, and cccDNA was further linearized by EcoRI digestion (lanes 1–2). The ssDNA species was subsequently digested by PSAD (lane 4). In addition to eliminating ssDNA, PSAD digestion resulted in smeared HBV DNA signals. These smeared signals were not apparent in Hirt DNA treated with Exo I/III but were detectable following T5 exo (lanes 5–6). The nature of the smeared signals is unclear but might originate from the incompletely digested HBV transgene-containing host DNA fragments in heterogeneous lengths.

While Exo I/III and T5 exo digestions effectively removed DP-rcDNA, both treatments, especially T5 exo, also markedly reduced cccDNA signals (Fig. 3A, lower panel, lanes 5–6). Densitometric quantification showed that the EcoRI-linearized cccDNA signals following Exo I/III and T5 exo digestion were 83.7 and 46.6%, respectively, of the cccDNA signal obtained without enzymatic treatment (lane 2). These findings are consistent with previous studies (10, 12). This over-digestion is confirmed by decreased total HBV DNA copy number by HBV1655 qPCR and lower cccDNA levels measured by cross-gap cccDNA qPCR assay compared with DNA treated by 85°C heat denaturation, followed by PSAD digestion or DNA quantified by BSC-cccDNA assays (Fig. 3B). Notably, cccDNA quantities, 1,102,500 (by BSC-cccDNA v55) and 892,500 (by BSC-cccDNA v300) represent approximately 10% of total untreated HBV-DNA (10,700,000), consistent with the Southern blot result (Fig. 3A, lower panel, lane 2).

### Detection of cccDNA in liver biopsy samples using BSC-cccDNA assays

Next, we explored the application of BSC-cccDNA assay to liver biopsy samples. Tissue total DNA isolated from CHB patients with a wide range of serum viral load DNA (3–8 log IU/mL; Table 3) was first quantified using HBV1655 qPCR assay, and then subjected to BSC-cccDNA v55 and v300 qPCR assays.

**TABLE 3:** Detection of cccDNA in liver tissue biopsy by BSC-cccDNA qPCR (v55 and v300) assays

ID	Serum viral load (IU log/mL)	HBV 1655^*a*^ (cp/million cells)	BSC-cccDNA v55 (cp/million cells, mean ± SD)	BSC-cccDNA v300 (cp/million cells, mean ± SD)
L1	7.10	2.67 E8	(4.56 ± 0.60) E6	(1.39 ± 0.004) E6
L2	8.40	3.55 E8	(4.21 ± 0.32) E6	(4.33 ± 0.004) E6
L3	7.70	5.54 E8	(8.06 ± 1.20) E6	(8.71 ± 0.04) E5
L4	5.69	1.86 E7	(7.51 ± 1.36) E5	(2.77 ± 0.08) E5
L5	4.77	1.96 E8	(1.50 ± 0.26) E7	(2.82 ± 0.16) E5
L6	4.16	5.04 E6	(3.24 ± 0.58) E5	(1.06 ± 0.04) E5
L7	6.70	5.44 E7	(3.65 ± 0.78) E6	(6.36 ± 0.26) E5
L8	6.31	2.40 E8	(7.26 ± 2.58) E6	(3.89 ± 0.32) E5
L9	8.01	6.67 E8	(1.52 ± 0.72) E7	(7.93 ± 0.002) E6
L10	2.66	5.81 E7	(3.71 ± 1.04) E5	BLOD
L11	8.12	6.20 E7	(2.64 ± 0.34) E6	(2.20 ± 0.002) E6
L12	8.48	1.46 E8	(2.70 ± 0.94) E6	(1.15 ± 0.04) E6
L13	8.67	8.74 E7	(3.89 ± 1.34) E6	(1.84 ± 0.002) E6
L14	6.57	4.62 E7	(1.77 ± 0.46) E5	(3.43 ± 0.002) E6
L15	8.23	2.20 E7	(1.13 ± 0.71) E6	(9.48 ± 0.14) E5
L16	3.31	2.19 E7	(1.08 ± 0.84) E6	(9.46 ± 0.26) E4

^*a*^ HBV input quantities were determined by HBV 1655 qPCR assays. Samples were analyzed in duplicate, and results were accepted when the two Cp values differed by less than 0.5 cycles. Samples with Cp values differing by more than 0.5 cycles were reanalyzed in triplicate, and the mean was calculated from the measurements differing by less than 0.5 cycles. BLOD: below the limit of detection.

As expected, in most samples, lower cccDNA copy numbers were obtained using BSC-cccDNA v300 assay as compared with the short-amplicon BSC-cccDNA v55 assay, reflecting the differences in amplicon size. Notably, however, in sample L14, substantially higher cccDNA levels were detected by BSC-cccDNA v300 assay than by BSC-cccDNA v55 assay. To investigate this discrepancy, PCR products from sample L14 generated by HBV 1526_1949 (Table S2) were subjected to Sanger sequencing. Sequence analysis revealed two point mutations (1811G > T and 1814C > T) within the reverse primer-binding site of the BSC-cccDNA v55 assay. This double mutation likely impaired primer annealing, thereby explaining the markedly lower signal obtained with the BSC-cccDNA v55 assay in this sample.

**Fig 4:**
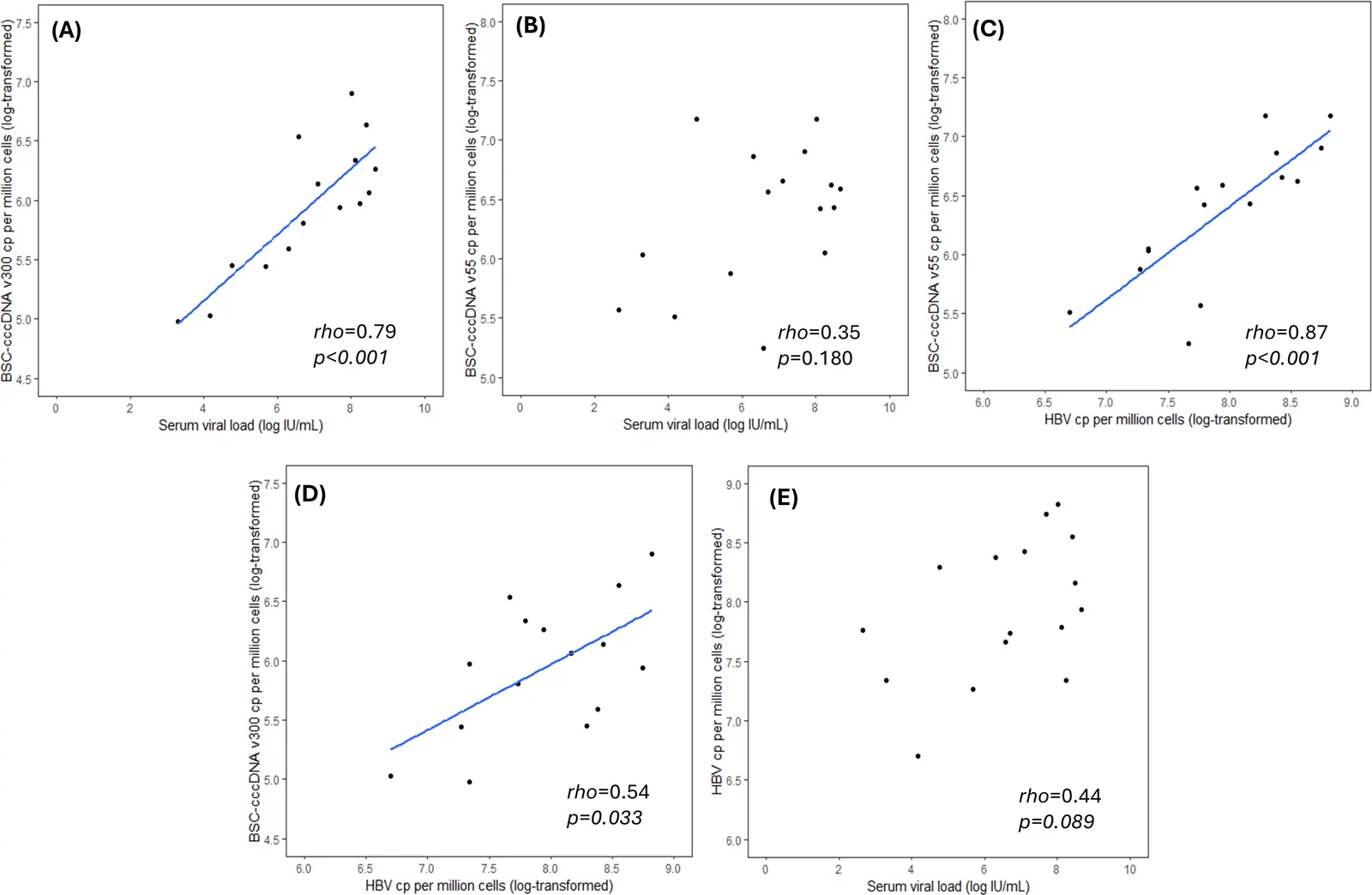
Correlation of intrahepatic BSC-cccDNA quantity with serum viral load and intrahepatic HBV DNA from liver biopsy samples. Intrahepatic HBV DNA was quantified by HBV1655 qPCR, and cccDNA levels were measured by BSC-cccDNA v55 (55 bp) and v300 (300 bp) qPCR assays. Dot plots were displayed for correlation analysis between cccDNA measured by v300 and serum viral load (**A**), v55 and serum viral load (**B**), v55 and intrahepatic total HBV DNA (**C**), v300 and intrahepatic total HBV DNA (**D**), and intrahepatic total HBV DNA and serum viral load (**E**). Spearman’s rank correlation analysis was performed for each comparison, and corresponding ρ (rho) and *P* values are indicated in the panels.

It was of interest to examine whether cccDNA quantities measured by BSC-cccDNA assays correlate with serum viral load. Spearman’s correlation analysis was performed between serum HBV DNA levels and intrahepatic cccDNA quantified by either the BSC-cccDNA v55 or BSC-cccDNA v300 assays. As shown in Fig. 4, a strong correlation was observed between serum viral load (log IU/mL) and cccDNA measured by BSC-cccDNA v300 assay (Fig. 4A, *ρ* = 0.79, *P* < 0.001), but not with cccDNA measured by the BSC-cccDNA v55 assay (Fig. 4B, *ρ* = 0.35, *P* = 0.18). Interestingly, intrahepatic total HBV DNA levels were strongly correlated with cccDNA measured by the BSC-cccDNA v55 assay (Fig. 4C, *ρ* = 0.87, *P* < 0.001) and only moderately correlated with cccDNA measured by BSC-cccDNA v300 (Fig. 4D, *ρ* = 0.54, *P* = 0.03). The intrahepatic HBV DNA was not found to be correlated to viral load (Fig. 4E, *ρ* = 0.44, *P* = 0.089). Collectively, these findings suggest that BSC-cccDNA v300 assay enables quantification of intrahepatic cccDNA in clinical liver biopsy specimens and shows strong correlation with serum viral load, supporting its potential utility in translational and clinical HBV studies.

## DISCUSSION

In this study, we evaluated a commercially available, patented approach for cccDNA quantification, including both the BSC-cccDNA v55 (55-bp amplicon) and v300 (300-bp amplicon with an internal probe) qPCR assays (Fig. 1). Using defined synthetic srcDNA substrates and cell culture-derived cytoplasmic HBV core DNA, we demonstrate that the BSC-cccDNA assays maintain high specificity for cccDNA, even in the presence of up to 10⁹ copies of srcDNA or 10⁵ copies of HBV core DNA (Table 1). When applied to DNA isolated from cell culture models and clinical liver biopsy samples, the BSC-based assays provided a sensitive and specific method for cccDNA quantification while minimizing rcDNA-derived false-positive amplification.

Analytical characterization revealed distinct performance profiles for the two BSC-cccDNA assays. The short-amplicon BSC-cccDNA v55 assay achieved a detection limit of approximately 10 copies with broad linearity, whereas the longer-amplicon BSC-cccDNA v300 assay, although capable of detecting as few as 10 copies, required ≥100 copies for reliable quantification (Fig. 2). These differences are consistent with the known impact of amplicon length and template integrity on PCR sensitivity. We and others have previously shown that, when working with fragmented DNA templates, the distance between primer binding sites significantly influences assay sensitivity (15, 21–25). Bisulfite conversion is known to compromise DNA integrity by converting stable double-stranded DNA into more labile single-stranded DNA and by introducing strand breaks, resulting in fragmented templates. Consequently, assay performance is particularly sensitive to amplicon length under these conditions. This was effectively mitigated by using a short (55-bp) amplicon, enabling the BSC-cccDNA v55 assay to achieve sensitivity comparable to that of dPCR evaluated in this study (Table 2). In contrast, the v300 assay provides enhanced specificity by incorporating an internal probe and requiring intact minus-strand continuity across a larger viral DNA region. This design may be advantageous in applications where maximal specificity is prioritized over sensitivity.

In addition to compromising DNA integrity, bisulfite conversion alters DNA sequence depending on the methylation status of cytosines, which can vary in clinical samples. To ensure assay sensitivity across diverse methylation states of cccDNA templates, the primer set was designed as a cocktail of oligonucleotides capable of annealing to bisulfite-converted sequences derived from either methylated or unmethylated cytosines. Because the primers were engineered to recognize both methylated and unmethylated variants within the primer-binding regions, amplification can still occur, even when bisulfite conversion is incomplete. This design consideration is important given that bisulfite conversion efficiency varies across technologies, with reported rates ranging from approximately 98% to 99.5% (26).

Encouragingly, analysis of 16 liver biopsy samples demonstrated that the cocktail primer design enabled quantification of intrahepatic cccDNA (Table 3). Notably, cccDNA levels measured by the BSC-cccDNA v300 assay showed a strong correlation with serum viral load (*ρ* = 0.79, *P* < 0.001), whereas cccDNA quantified by the short-amplicon BSC-cccDNA v55 assay did not (*ρ* = 0.35, *P* = 0.18). These findings suggest that longer-amplicon with an internal probe detection may provide more specificity in clinical samples, while the short-amplicon assay, although more sensitive, may also detect partially fragmented templates or non-cccDNA species, such as viral double-stranded linear DNA (dslDNA) and/or integrated DNA (iDNA), which were not systematically evaluated in this study.

Consistent with the impact of amplicon size on assay sensitivity, higher cccDNA quantities were measured by the v55 assay compared with the v300 assay in all clinical samples, except sample L14. Subsequent Sanger sequencing identified two nucleotide mismatches within one primer-binding site of the v55 assay in this sample. This finding provides at least a partial explanation for the observed discrepancy and underscores the influence of HBV sequence variability on primer-dependent amplification efficiency. These results further suggest that the use of both the BSC-cccDNA v55 and v300 qPCR assays may help mitigate the effects of HBV genetic variation in clinical samples. Application of the BSC-cccDNA assays to liver biopsy specimens also demonstrated their feasibility in clinical material spanning a broad range of serum viral loads.

Direct comparison with commonly used exonuclease-based strategies highlights important limitations of enzymatic pretreatment approaches. Although Exo I/III and T5 exo effectively reduced rcDNA and other DNA species with accessible 3′ and 5′ ends, respectively, both treatments also substantially diminished detectable cccDNA signals, as demonstrated by Southern blot, HBV1655 qPCR, and conventional cross-gap PCR (Fig. 3). This reduction likely results in underestimation of the absolute cccDNA levels following enzymatic digestion. Importantly, Southern blot quantification estimated that cccDNA accounted for approximately 8% of total HBV DNA in untreated HepAD38 Hirt DNA (Fig. 3A), which was highly consistent with the values obtained by BSC-cccDNA v55 (10.3%) and v300 (8.3%) assays. This agreement supports the accuracy of the BSC-based approach for measuring native cccDNA levels without the need for nuclease pretreatment. The reason behind such discrepancies across different assays is unknown, but likely due to the fact that the DNA recovery rate may vary considerably depending on pretreatment and column purification steps. Notably, the heat treatment at 85°C alone resulted in an approximately fivefold total DNA loss in qPCR quantitation without altering the Southern blot detection. Together, these findings suggest that enzymatic pretreatment may alter the apparent cccDNA proportion, whereas the BSC-based approach more closely reflects the native distribution of HBV DNA species.

Several limitations of this study should be acknowledged. Although the BSC-cccDNA assay demonstrates high specificity in distinguishing cccDNA from rcDNA, further investigation is needed to define its specificity relative to other non-rcDNA, non-cccDNA HBV DNA species, including the heterogeneous immature HBV DNA replication intermediates and iDNA, especially when dealing with total DNA samples rather than Hirt DNA. Additionally, as demonstrated in the small cohort of 16 liver biopsy samples, as expected, HBV sequence heterogeneity exists in clinical specimens. Despite the use of a cocktail primer design to accommodate methylation variability and sequence diversity, nucleotide variation within primer-binding regions may still affect amplification efficiency and quantification accuracy. Lastly, while the observed correlations with viral markers support the biological relevance of the assay, validation in larger and independent cohorts will be necessary to confirm these findings and further define the clinical utility of the BSC-cccDNA approach.

## MATERIALS AND METHODS

### Construction of cccDNA control plasmid (H100)

A plasmid containing a monomeric hepatitis B virus (HBV) genome was constructed and used as a cccDNA control. The EcoRI-linearized HBV genotype D genome (3,182 nt; GenBank accession no. U95551.1) was cloned into the EcoRI site of the pGEM-3Z vector (Promega, Cat# P2151), giving rise to pGEM-3Z-H100 (referred to as H100). The resulting plasmid was propagated in *E. coli* and purified using standard plasmid preparation procedures. H100 served as a positive control template for BSC-cccDNA assay development and validation.

### Cell culture, HBV infection, and test sample preparation

The tetracycline(tet)-inducible HBV stable cell line HepAD38 cells were maintained in Dulbecco’s Modified Eagle’s Medium/Ham’s F-12 (DMEM/F-12, Cytiva) supplemented with 10% fetal bovine serum (FBS), 100 U/mL penicillin, 100 μg/mL streptomycin, and 1 μg/mL tet, as previously described (16, 27). To induce HBV replication and cccDNA formation, tet was withdrawn from the culture medium. Cells were cultured in tet-free medium for 7 days prior to HBV core DNA extraction and for 14 days prior to Hirt DNA extraction. Primary human hepatocytes (PHHs) were obtained from ScienCell Research Laboratories (Cat# 5200) and cultured in hepatocyte medium (Cat# 5201) supplemented with 5% FBS (Cat# 0025), 1% hepatocyte growth supplement (Cat# 5252), and 1% penicillin/streptomycin solution (Cat# 0503). PHHs were infected by HBV and subjected to Hirt DNA extraction as described previously (28).

### HBV cccDNA quantification by digital PCR after T5 and T7 exonuclease digestion

HBV cccDNA in Hirt DNA isolated from infected PHH cells was quantified using digital PCR (dPCR) with the Clarity Digital PCR System (JN Medsys) according to the manufacturer’s instructions. Next, 1 µg of DNA extracted using a modified Hirt method was treated with 5 units each of T5 and T7 exonucleases (NEB, Cat# M0363 and M0263, respectively) at 30°C for 90 min to digest any contaminating rcDNA (11). This was followed by a 99°C incubation for 5 min to halt the enzymatic reactions. After digestion, the cccDNA in the samples was measured through dPCR. The sequences of the cccDNA-specific PCR primers and the TaqMan probe are provided in Table S1. The cccDNA template was divided into a multi-well PCR chip, allowing the PCR to be separated into thousands of nanoliter sub-reactions, each containing at most a single copy of DNA. The dPCR protocol followed a previously established method (29). Following dPCR, the images of the PCR chips were scanned and transformed into scatter plots showing the FAM dye signals from the hybridization probe during the PCR process. The number of cccDNA copies present in the sample was then calculated.

### HBV DNA PCR assays

Various PCR assays were used in this study for specified purposes, as summarized in Table S1. Briefly, HBV DNA quantity for plasmid H100 cccDNA control, core, or Hirt DNA was quantified by HBV1655 qPCR assay. The quantity of srcDNA was measured by the HBV 1778 qPCR assay. cccDNA quantification after nuclease pretreatment was measured by either dPCR as described above or cross-gap qPCR (8, 9, 12) as specified.

### BSC-cccDNA assays

To quantify cccDNA in the presence of rcDNA, we used the commercial HBV BSC-cccDNA Quantification Kit v300 (JBS Science, Inc., Cat# qPCR-021-v300) and v55 (Cat# qPCR-021-v55), which were developed based on a patented cccDNA detection technology (13). DNA samples first underwent bisulfite conversion treatment using JBS BSC Kit (Cat# JBS-08878), which renders the two DNA strands non-complementary. Because sodium bisulfite is relatively unstable in liquid solution, storage after reconstitution can reduce BSC efficiency. In the JBS BSC Kit, sodium bisulfite is supplied in powder form for stability concern. To perform the BSC, we first reconstituted the BSC reagents according to the manufacturer’s specifications. Following BSC, the DNA samples were either subjected immediately to HBV BSC-cccDNA quantification using the v55 and v300 kits according to the manufacturer’s specifications or stored at −80°C in aliquots to avoid repeated freeze/thaw cycles. The JBS BSC-cccDNA qPCR assays were designed to amplify bisulfite-treated minus strand across the nick adjacent to the DR1 site present in rcDNA, as illustrated in Fig. 1. The same forward primer sequences were used for both the v55 and v300 assays, whereas the reverse primer sequences differ, as indicated in Fig. 1C. The primers consist of cocktails of oligonucleotides designed to ensure coverage of both methylated and unmethylated DNA templates across multiple HBV genotypes. Primer sequence homology analyses were performed against HBV sequences downloaded from HBVdb (https://hbvdb.lyon.inserm.fr/HBVdb/HBVdbIndex). As summarized in Table S2, the v55 assay demonstrated overall sequence identities of 86% and 83% to methylated and unmethylated sequences, respectively, across all listed HBV genotypes. In comparison, the v300 assay showed overall sequence identities of 75% and 74% to methylated and unmethylated sequences, respectively, across all genotypes, except for genotypes F, G, and H.

### Specificity and sensitivity assessment

**Fig 5:**
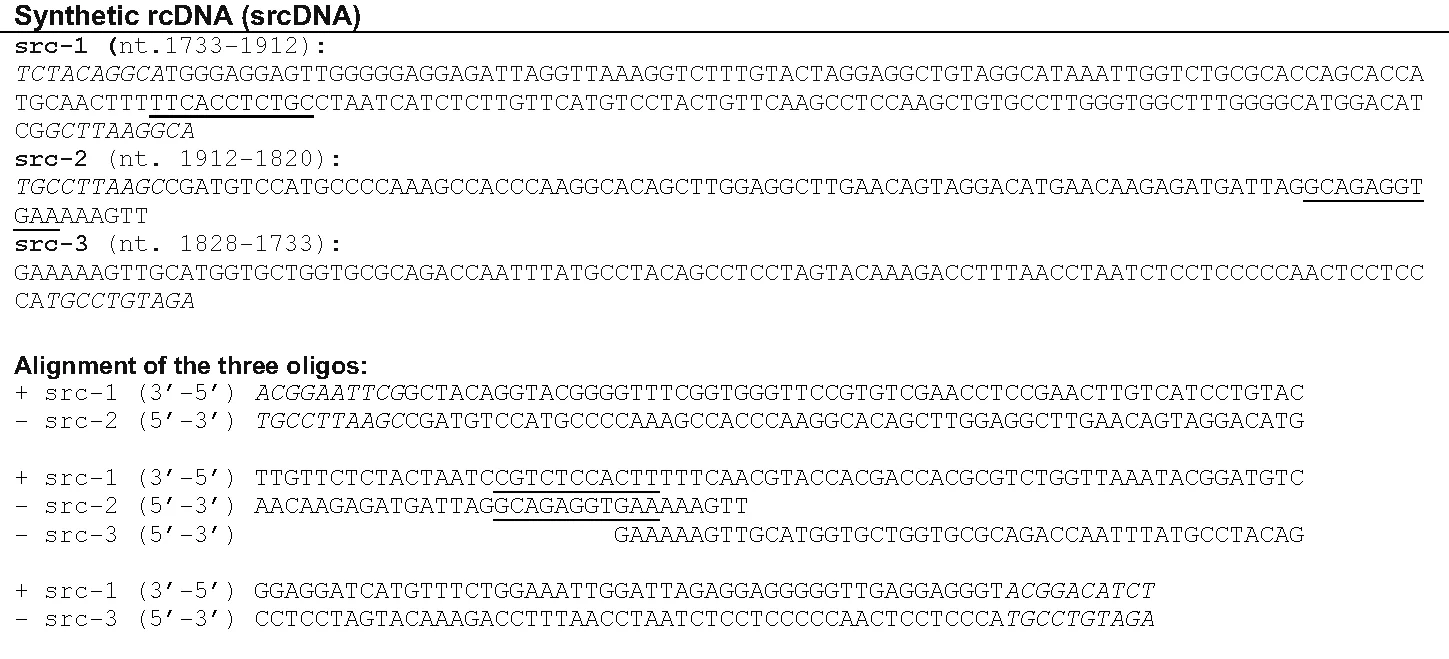
Sequences and structure of synthetic rcDNA (srcDNA). DR1 is indicated by underlining; non-HBV tag sequences are in italic; + and – denote the plus and minus strands, respectively; strand direction is indicated by either 3′−5′ or 5′−3′.

To assess the specificity and sensitivity of cccDNA assays, we used H100 plasmid as a cccDNA control and a synthetic rcDNA (srcDNA) fragment (nt 1733–1912) as an rcDNA control for the BSC-cccDNA v55 qPCR assay. To prepare for srcDNA, three oligos src-1 (nt. 1733–1912), src-2 (nt. 1912–1819), and src-3 (nt. 1828–1733) sequences listed in Fig. 5 were synthesized by IDT (https://www.idtdna.com/). After resuspension in IDTE buffer (IDT, Cat# 11-05-01-05), the three oligos were pooled equimolarly and heated at 56°C for 15 min to form the srcDNA fragments containing the nick and flap on the minus strand (30). The srcDNA substrates were then aliquoted, quantified by HBV 1778 qPCR assay, and stored in −80°C freezer until usage.

Purified cytoplasmic HBV core DNA containing all the capsid-associated viral DNA replicative intermediates, including the native relaxed circular DNA (rcDNA), was prepared from induced HepAD38 cells.

The concentrations of all DNA standards were determined using a Qubit fluorometer (Thermo Fisher Scientific, Waltham, MA, USA) and by the HBV 1655 qPCR assay or HBV 1778 qPCR assay for srcDNA. The primer sequences and PCR conditions for these HBV qPCR assays are detailed in Table S1.

To evaluate the assay’s sensitivity, H100 plasmid was serially diluted with human genomic DNA (Promega, Cat# G3041) or sonicated human gDNA at 0.5 ng/μL. The series was then bisulfite treated and used as input for BSC-cccDNA assays. The BSC-cccDNA assays can consistently detect 100 copies of input H100 plasmid in either 10 μL or 20 μL qPCR reaction volume. It can detect as low as 10 copies of input H100 plasmid, but care must be taken to prevent sample contamination by HBV DNA, while background human gDNA gives no amplification after bisulfite conversion.

To evaluate the assay’s specificity, serial dilutions of srcDNA and purified core DNA samples were made with and without 100 copies of H100 plasmid, respectively. The four series were then bisulfite treated and used as input samples for the BSC-cccDNA assay.

### Exonuclease pretreatments and Southern blot hybridization

HBV cytoplasmic core DNA or Hirt DNA samples were heat denatured at 85°C for 5 min and subsequently digested with PSAD (Bioresearch Technologies, E3101K) for 16 h at 37°C, Exo I/III (New England Biolabs (NEB), Cat# M0293 and M0206), or T5 exo (NEB, Cat# M0663), as previously described (9–11). All reactions were performed in the corresponding manufacturer-recommended buffers. Enzymatic reactions were terminated by heat inactivation according to the manufacturers’ manuals. Following exonuclease digestion, samples were either further digested with EcoRI to linearize cccDNA for Southern blot analysis (7, 30) or purified using QIAquick PCR Purification Kit (Qiagen, Cat# 28106) for qPCR analysis.

### Liver biopsy DNA analyses

Sixteen archived treatment-naïve CHB patient liver biopsy DNA samples described in a previous study (31) were used for HBV total DNA and cccDNA analyses, as indicated in Table 3.

### Statistical analysis

Spearman’s correlation analysis was performed between serum HBV DNA levels and intrahepatic HBV DNA quantified by HBV 1655 qPCR and cccDNA quantified by either the BSC-cccDNA v55 or BSC-cccDNA v300 assays.

## Data Availability

All data supporting the findings of this study are available within the article and its supplemental material.

## SUPPLEMENTAL MATERIAL

Table S1 jvi.00961-26-s0001.docx Oligonucleotides and reaction conditions.

Table S2 jvi.00961-26-s0002.xlsx Primer homology analysis.

ASM does not own the copyrights to Supplemental Material that may be linked to, or accessed through, an article. The authors have granted ASM a non-exclusive, world-wide license to publish the Supplemental Material files. Please contact the corresponding author directly for reuse.
