# Supplementary material for: A novel highly specific qPCR assay for distinguishing HBV cccDNA from rcDNA following bisulfite pretreatment
Source: J Virol. 2026 Aug 14;100(9):e00961-26. doi: 10.1128/jvi.00961-26 (PMC13595978; doi:10.1128/jvi.00961-26)
Supplement: Supplementary file 1 [file jvi.00961-26-s0001.docx]

**Supplemental Table 1. Oligonucleotides and reaction conditions.**

| **Primers** | **sequences** | **Purpose and PCR condition** |
| --- | --- | --- |
| HBV 1655_F (1655-1673) | *TGTAAAACGACGGCCAGT***CATAAGAGGACTCTTGGAC** | HBV DNA quantification; 95°C 5 mins, then 95°C 10s, 60°C 10s, 72°C 10s for 45 cycles. |
| HBV 1655_R (1703-1682) | *CAGGAAACAGCTATGAC***CCTCAAGGTCGGTCGTTGACAT** |  |
| HBV 1778_F (1778-1800) | *CAGGAAACAGCTATGACCAACCTTGGAT***GGAGGCTGTAGGCATAAATTGGT** | srcDNA quantification; 95°C 5 mins, then 95°C 10s, 65°C 10s, 72°C 10s for 45 cycles. |
| HBV 1778_R (1852-1829) | *TGTAAAACGACGGCCAGTAGGAACCTTC***AACAWGAGATGATTAGGCAGAGGT** |  |
| HBV 1526_1949_F (1526-1543) | *^#^CAGGAAACAGCTATGACC***GCGCACCTCTCTTTACGC** | PCR product for Sanger sequencing; 95°C 10 mins, then 95°C 15s, 61°C 60s for 45 cycles. 4°C hold. |
| HBV 1526_1949_R (1949-1925) | **AGTAACTCCACAGWAGCTCCAAATT** |  |
| Cross-gap_F (1553-1572) | **GTCTGTGCCTTCTCATCTGC** | cccDNA quantification after removal of rcDNA; 95°C 5 mins, then 95°C 15s, 61°C 60s for 45 cycles. 4°C hold. |
| Cross-gap_R (1949-1925) | **AGTAACTCCACAGWAGCTCCAAATT** |  |
| Cross-gap_Probe (1865-1894) | FAM-**TTCAAGCCTCCAAGCTGTGCCTTGGGTGGC-**TAMRA |  |
| dPCR_F  (1562–1579) | **TTCTCATCTGCCGGACCG** | cccDNA quantification after removal of rcDNA; 95*°*C 5 min and then of 95*°*C 50s, 55*°*C 100s for 40 cycles and 70*°*C 5 min, 4°C hold. |
| dPCR_R  (1883–1864) | **CACAGCTTGGAGGCTTGAAC** |  |
| dPCR_probe  (1836–1855) | FAM-**CCTAATCATCTCTTGTTCAT**-MGB |  |

HBV reference GenBank accession # U95551.1; non-HBV tag sequence: italic, HBV sequence: bold, ^#^non-HBV M13 reverse tag *italicized*.
